# Biological characterization of novel *Escherichia coli* O157:H7 phages and their bacteriostatic effects in milk and pork

**DOI:** 10.3389/fmicb.2025.1516223

**Published:** 2025-02-06

**Authors:** Qinghai Ren, Zhiwei Wang, Yichen Ge, Yucui Huang, Wei Zhang, Chunxue Liu, Yubao Li, Shengliang Cao

**Affiliations:** ^1^College of Agriculture and Biology, Liaocheng University, Liaocheng, Shandong, China; ^2^College of Veterinary Medicine, Nanjing Agricultural University, Nanjing, China; ^3^Anyou Biotechnology Group Co., Ltd., Suzhou, China

**Keywords:** *Escherichia coli* O157:H7, phage, biological characteristics, genomic analysis, antibacterial effect

## Abstract

Foodborne bacteria, particularly *Escherichia coli* (*E. coli*) O157:H7, are significant contributors to foodborne illnesses, with antibiotic overuse exacerbating the issue through the emergence of multidrug-resistant strains. This study investigated the potential of *E. coli* phages in food safety, examining their biological traits and bacteriostatic properties. Two phages (vB_EcoP_SD2, vB_EcoP_SD6) of *E. coli* O157:H7 were isolated from slaughterhouse sewage and characterized for morphology, genomic composition, phage phylogenetic tree, optimal multiplicity of infection (MOI), one-step growth curve, thermal and pH stability and antibacterial efficacy. The optimal MOIs of vB_EcoP_SD2 and vB_EcoP_SD6 was 0.1 and 0.01, and temperature range for maintaining activity was 4°C to 55°C. The host range of vB_EcoP_SD2 and vB_EcoP_SD6 was 65% (13/20) and 55% (11/20), which was partially complementary to each other (75%, 15/20). Notably, vB_EcoP_SD2 displayed a latent period of 10 min, a burst period of 80 min, and a burst volume of 80 PFU per cell, while vB_EcoP_SD6 had a burst volume of 10 PFU per cell. Comprehensive whole-genome analysis confirmed two phages has no presence of pathogenic factors or resistance genes. Genomic comparisons suggest vB_EcoP_SD2 and vB_EcoP_SD6, respectively, constituted a novel member of a new genus, Justusliebigvirus genus and Kayfunavirus genus which genome, respectively, was found to be 1,49,066 bp, 40,202 bp long with an average GC content of 37.5 and 49.8%. The phages effectively inhibited host bacteria in LB broth for at least 6 h and showed promise in inhibiting bacteria in milk and pork, which indicated that the two phages exhibited a favorable bacteriostatic effect on milk and pork within the first 6 h under the optimal MOI. In the milk bacteriostasis experiment, vB_EcoP_SD2 could reduce bacteria by 3.16 × 10^4^ CFU/mL, and vB_EcoP_SD6 could reduce bacteria by 1.05 × 10^4^ CFU/mL. Phage vB_EcoP_SD2 decreased bacteria by 1.14 × 10^4^ CFU/mL, and vB_EcoP_SD6 decreased bacteria by 2.04 × 10^3^ CFU/mL in the pork. There was no disparity in bacteriostatic effect of different MOI within the first 6 h, but bacteriostatic effect of all groups still remained different from that of the control group. This study indicates the two phages possess excellent biological characteristics, thereby providing a theoretical foundation for the subsequent development of natural fungicides.

## 1 Introduction

Foodborne bacteria represent the primary causative agent of foodborne illnesses and food poisoning *Escherichia coli* O157:H7 is a significant foodborne pathogen capable of causing gastroenteritis in humans (Mead et al., 1999), posing a substantial threat to global public health (Bumunang et al., 2024). Increasing antimicrobial resistance (AMR) has contributed to the severity of *E. coli* O157:H7 infections (Ammar et al., 2021). Pathogenic bacteria have the capacity to cause extensive outbreaks of infectious diseases in livestock and poultry by contaminating their food and water supplies. Furthermore, the ingestion of these bacteria can result in intestinal infections, which presents a significant risk to both food safety and human health (Chai et al., 2019). *Escherichia coli* (*E. coli*), *Staphylococcus aureus*, and *Salmonella* are examples of common bacteria that have been identified as the etiological agents of foodborne illnesses (Zhang et al., 2020). *E. coli* is a significant foodborne pathogen that is highly prevalent in the environment, river water, animals, and plants. It is responsible for producing a number of ailments, including urinary tract infections, diarrhea, and bloodstream infections. *β*-lactam antibiotics, including broad-spectrum cephalosporins and carbapenems, are commonly used as the initial therapy for multi-resistant Gram-negative bacteria (Mellata, 2013; Lima et al., 2020). The overuse of antibiotics has resulted in the emergence of multidrug-resistant *E. coli*, which has made it challenging to contain in livestock and poultry production.

The physical methods frequently utilized in food production to control foodborne pathogenic bacteria encompass steam, dry heat, and ultraviolet radiation. Thermal killing technology represents a conventional method of physically sterilizing food. However, the high temperature used in this process results in significant alterations to the color and flavor of the food, as well as a considerable reduction in its nutritional value. Conversely, the implementation of cold sterilization technology carries the potential for recontamination (Leon-Velarde et al., 2019). Furthermore, chemical treatment methods, including the use of fungicides and preservatives, are employed. The application of these two approaches results in a modification of the sensory characteristics of the foodstuff, which in turn elicits a customer aversion due to the non-compliance of the food product with the environmentally friendly food standards (Atterbury, 2009). Additionally, several methods of preventing the growth of harmful bacteria that cause foodborne illnesses are not directly applicable to fresh produce, such as fruits and vegetables, or ready-to-eat items.

Phages are bacteria viruses that specifically infect and lyse bacteria, which constitute the most abundant biological entities in the biosphere, with an estimated 10^31^ phage particles (Harada et al., 2018). In recent years, phages have garnered significant attention as a biological control agent for reducing or eliminating pathogenic microorganisms (Vikram et al., 2020). Indeed, numerous studies have highlighted the potential of phages as a viable alternative to conventional antimicrobial strategies. Phages exhibit a high degree of specificity for their bacterial hosts, serve as natural antagonists of bacteria in various ecosystems, and do not interfere with other microbiota present within the human body (Navon-Venezia et al., 2017). Given the escalating global concern regarding foodborne pathogens as a significant public health threat, the exploration and advancement of phage applications is poised to become a focal point of scientific inquiry. In the present investigation, we successfully isolated and purified phages targeting *E. coli* from untreated sewage sourced from pig farms, evaluated their tolerance to varying temperatures and pH levels, assessed their adsorption rates, and delineated their one-step growth kinetics, while also examining the phage’s biological morphology utilizing transmission electron microscopy. Subsequently, we performed comprehensive genomic and phylogenetic analyses of the isolated phages, alongside functional annotations pertaining to their taxonomic classification. Ultimately, we assessed the *in vitro* antimicrobial efficacy of the phages as well as their therapeutic influences on dairy milk and pork meat, thereby establishing a foundational basis for further research and innovative strategies aimed at mitigating *E. coli* contamination in food commodities.

## 2 Materials and methods

### 2.1 *E. coli* strain O157:H7 isolation, growth condition, and PCR identification

In 2023, a specific strain of *E. coli* which serotype is O157:H7, designated EC2318, was successfully isolated from biobanked clinical samples routinely collected from pig farms and slaughterhouses in Shandong Province, China. The isolation of EC2318 strains was conducted following the method previously established by our laboratory. Purification was performed three times on McConkey’s medium by three-zone striation method, and 16S rDNA was verified by PCR for that region (FP: 5′ -CGTTTCTACCGCAGAGTTG-3’ and RP: 5’-GTCATCTGTGCCAGGGTC-3′). After PCR, the amplicon size was confirmed by gel electrophoresis, and the DNA was extracted from the 1% agarose gel, and sequenced at Tsingke Biotech Co., Ltd., China. The obtained sequence was checked by BLASTn against the rRNA/ITS database at the National Center for Biotechnology Information (NCBI) portal.

### 2.2 Assessing drug susceptibility and virulence gene in *E. coli* strain EC2318

Antimicrobial susceptibility testing of bacterial isolates was performed according to the Clinical and Laboratory Standards Institute (CLSI) 2019 recommendations (Cheng et al., 2019). Overnight-grown colonies were aseptically suspended in sterile isotonic saline to achieve a visual opacity equivalent to the 0.5 McFarland standard. The bacterial suspension was then aseptically plated evenly on Mueller-Hinton agar (MHA) (Oxoid, United Kingdom). Antimicrobial susceptibility disks were strategically placed on the MHA. Agar plates were incubated at 37°C for 16 h. The antibacterial inhibition zones surrounding the disks were measured on three separate occasions, and the average diameter was calculated and interpreted as either susceptible, intermediate, or resistant according to CLSI guidelines. The 12 antimicrobials, classified into nine different antimicrobial categories, were amoxicillin (20 μg), ampicillin (10 μg), cefoxitin (30 μg), imipenem (10 μg), gentamicin (10 μg), amikacin (30 μg), ciprofloxacin (5 μg), sulfamethoxazole (300 μg), erythromycin (15 μg), tetracycline (30 μg), chloramphenicol (30 μg), and polymyxin B (300 IU). The susceptibility disks were purchased from Hangzhou Binhe Microbiological Reagent Co., Ltd. China. Isolates resistant to three or more antibiotic classes were classified as multidrug-resistant (MDR). In accordance with the recommendations of Kahlmeter et al. (2019), strains exhibiting resistance and those classified as intermediate were categorized as “non-susceptible,” with the suggestion to use the term “non-susceptible” instead of “insensitive.” Isolates that were non-susceptible to three or more antimicrobial agents were considered multidrug resistant (MDR) (Magiorakos et al., 2012). PCR was employed to detect the presence of *papC, tsh, fimC, ibeB, vat, yijp, ibeA, ompA, neuc, cva/cvi, iss, iroN, fyuA, iucD, irp2,* and *chuA* virulence genes (Supplementary Table S1) in *E. coli* (Wang et al., 2011). Drug susceptibility test and virulence gene detection were performed on 20 *E. coli* isolates.

### 2.3 Phage system isolation and purification process

The isolation and purification of the phages were carried out according to previously described methods (Viazis et al., 2011; Kazibwe et al., 2020), albeit with certain modifications. In summary, particulate components in the sample were eliminated by centrifugation at 1,000 *g* for 10 min at a temperature of 4°C. The enriched culture was then centrifuged at 7,000 *g* for 10 min at 4°C, followed by filtration of the supernatant through a 0.22-μm filter membrane (Millipore, United States) to remove bacterial contamination. The resulting filtrate was combined with host strain EC2318 (OD_600_ = 0.6–0.8) and incubated at 37°C with shaking at 120 rpm to facilitate phage enrichment. The enriched culture was then subjected to filtration. Equal volumes (100 μL) of the filtrate and the host strain EC2318 were mixed with melted semi-solid medium (0.7% agar), then applied to a lysogeny broth (LB) culture plate using the double agar overlay technique and incubated overnight at 37°C. Purified phages were successfully harvested after six rounds of plaque purification and stored at 4°C for subsequent analyses.

### 2.4 Phage morphology observation

The quantification of phage concentration was achieved through the utilization of the PEG precipitation technique (Viazis et al., 2011). A concentrated phage specimen was deposited onto a slide, and a segment of copper mesh was secured with precision tweezers. Subsequently, the mesh was positioned on top of the phage concentrate, and any excess liquid was absorbed with filter paper. Then, a droplet of 2% phosphotungstic acid (PTA, pH = 7) was introduced onto the copper mesh. And, the mesh was transferred onto blotting paper for a period of natural desiccation, lasting 10 min. The morphological characteristics of the phages were examined with a JEM1400 transmission electron microscope.

### 2.5 Phage lytic spectrum determination

The lytic spectrum of phages vB_EcoP_SD2 and vB_EcoP_SD6 was ascertained through the utilization of the spot test in conjunction with the double-agar overlay technique, as described in the previous survey (Sasikala and Srinivasan, 2016). In accordance with the clarity of the plaques, the following symbols were used to indicate the degree of lysis: “−” signified no lysis; “+” indicated weak lysis with a faint lysis spot; “++” represented moderate lysis with a clear lysis spot; and “+++” denoted strong lysis with a well-defined lysis zone.

### 2.6 Phage titer determination

The titer of the phage was determined through the use of the double-layer plate technique. The purified phage filtrate was subjected to a 10-fold serial dilution. A 10 mL centrifuge tube was filled with 200 μL of the phage dilution, along with 200 μL of the host bacterial solution. Subsequently, LB semi-solid medium at 55°C was added and mixed. This mixture was then plated onto an LB solid medium to create the double-layer plate. The plaques were then enumerated following a six-hour incubation period at 37°C. The phage titer (PFU/mL) was calculated by multiplying the number of phage plaques by the dilution factor.

### 2.7 Optimal phage infection complex identification

The optimal MOIs for the phages vB_EcoP_SD2 and vB_EcoP_SD6 were determined through the application of the methodology proposed (Lu et al., 2003), with certain modifications. The host strain EC2318 was prepared at a concentration of 10^7^ CFU/mL and subsequently incubated with diluted phage lysates in equal volumes (500 μL) at various MOI values, specifically 0.001, 0.01, 0.1, 1, 10, and 100. The host strain EC2318 was prepared at a concentration of 10^7^ CFU/mL and subsequently combined with the diluted phage at various MOI values, specifically 0.001, 0.01, 0.1, 1, 10, and 100. The resulting mixture was incubated at 180 rpm and 37°C for a period of 3 h, after which it was subjected to filtration through a 0.22-μm membrane filter. The determination of the phage titer was conducted via the double-agar overlay technique. The aforementioned experimental procedure was repeated on three occasions, with the highest titer of lysate indicating the optimal MOI for the phage.

### 2.8 One-step growth curve analysis for phage

In accordance with the methodology outlined by Sasikala and Srinivasan (2016), the host bacterial solution (10^7^ CFU/mL and 10^8^ CFU/mL) was combined with the phages separately according to their optimal MOI. The mixture was then incubated at 37°C for a period of 5 min in a water bath. Subsequently, the mixture was subjected to centrifugation at 12,000 rpm for 5 min, after which the supernatant was discarded. Subsequently, the pellet was washed twice with an equal volume of prewarmed LB medium at 37°C. Subsequently, 1 mL of prewarmed LB medium was added to resuspend the pellet. The suspension was then transferred to 100 mL of prewarmed LB medium and cultured in a 37°C shaker incubator. Samples were collected at 10-min intervals over a 120-min period in triplicate. The phage titer was determined using the double-layer agar plate method. A one-step growth curve was plotted with time (in minutes) on the *x*-axis and phage titer (in PFU/mL) on the *y*-axis. Phage burst size was calculated using the following formula: Lysis = (final phage titer − initial phage titer)/initial host bacteria number.

### 2.9 Phage temperature stability measurement

Seven 1-mL aliquots of phage (1 × 10^8^ PFU/mL) lysate were subjected to various temperatures in separate water baths set at 4, 25, 37, 45, 55, 65, and 75°C for a duration of 12 h (Oluwarinde et al., 2024). Subsequently, the phage titer at each temperature was determined via the double-layer agar plate method. To ensure the reliability of the results, the experiment was conducted in triplicate.

### 2.10 Phage pH stability measurement

The pH of the LB broth was adjusted to a range of 1–12 using 1 M HCl and 1 M NaOH. A volume of 100 μL of the phage stock, with a concentration of 10^8^ PFU/mL, was added to 900 μL of the pH-adjusted LB broth. The mixture was then thoroughly mixed and incubated at 37°C for 12 h. The phage titer at various pH levels was determined using the double-layer agar plate method, and the experiment was repeated in triplicate to ensure the reliability of the results.

### 2.11 Phage efficacy against bacterial host

The concentration dilution was performed using the optimal MOI between the phage and host bacteria. Specifically, 100 μL phage solution was mixed with 100 μL host bacterial culture, followed by incubation at 37°C. For the control group, an equivalent volume of sterile LB liquid medium was used in place of the phage-host mixture. Three replicate experimental groups were established and incubated at a consistent temperature of 37°C. Three parallel experimental groups were established and incubated at a consistent temperature of 37°C. The optical density (OD) at a wavelength of 600 nm was assessed at 2-h intervals over a continuous 24-h period.

### 2.12 Phage genome sequencing and analysis

Phage genomic DNA was extracted according to the E.Z.N.A.^®^ Viral DNA Kit (OMEGA, United States) kit instructions. A sequencing library was constructed using the TruSeq™ Nano DNA Sample Prep Kit (Illumina, United States). The genome sequencing was performed by Shanghai Linden Biotechnology Co., Ltd. on the Illumina novaseq6000 sequencing platform, resulting in the acquisition of complete genomes for two distinct phage groups.

GeneMarkS v4.28 (Besemer et al., 2001) was used to predict the open reading frame (ORF) of the phage genome and to manually retrieve the function of the ORF-encoding protein using BLASTp. Antimicrobial resistance genes (ARGs) and virulence factors were identified by querying the VFDB, CARD, and ResFinder databases (Liu et al., 2019; Bortolaia et al., 2020). Proksee (Grant et al., 2023) was used to visualize the phage genome. The genomes of phage vB_EcoP_SD2 and phage vB_EcoP_SD6 have been uploaded to the NCBI Genbank database. The major subunits of terminal enzymes and major capsid protein sequences of 46 strains of *E. coli* from different genera were downloaded from the NCBI protein database, and multiple sequences were compared by ClustalW. ModelFinder (Kalyaanamoorthy et al., 2017) was used to determine the optimal model, then IQ-TREE v2.2.6 (Nguyen et al., 2015) was used to build the maximum likelihood tree, bootstrap test was repeated for 1,000 times, and visualization was performed through iTOL (Letunic and Bork, 2021).

### 2.13 Phage as bacterial inhibitor in cow milk

The methodology proposed by Huang et al. (2018) was employed, whereby UHT pure milk (brand Yili) was aliquoted and divided into sterile test tubes, with every three samples forming one group. *E. coli* was cultivated until it reached the logarithmic growth phase, at which point it was diluted with sterile normal saline to a concentration of 10^7^ CFU/mL. The phage concentrations were generated at 10^5^ PFU/mL, 10^6^ PFU/mL, 10^7^ PFU/mL, 10^8^ PFU/mL, and 10^9^ PFU/mL. Phage concentrations of 10^5^ PFU/mL, 10^6^ PFU/mL, 10^7^ PFU/mL, 10^8^ PFU/mL, and 10^9^ PFU/mL were used in conjunction with MOIs of 0.01, 0.1, 1, 10, and 100, respectively. An additional tube containing a sample was enriched with 100 μL of sterile PBS to serve as a negative control. The specimens were placed in a temperature-regulated chamber maintained at a constant temperature of 25°C. At designated time intervals (0, 1, 3, 6, 9, and 12 h), 20 μL samples were collected and distributed onto LB solid medium plates. Prior to sampling, the plates were uniformly agitated, with each time point conducted in triplicate. Bacterial enumerations were conducted after 8 h of incubation by the plate counting method.

### 2.14 Phage as bacteriostatic agent in pork meat

In accordance with the methodology established by Huang et al. (2018), the fresh pork meat of tenderloin was meticulously sliced into sections approximately 0.2 cm in thickness, with each slice weighing 1 g. The pork meat sections were then subjected to a boiling process in water for a duration of 3 min, which effectively eradicated any bacteria. Then, the pork meat sections were partitioned into 36 distinct portions weighing 1 g, with each portion being systematically placed into a sterile culture dish. An aliquot of 100 μL of *E. coli*, characterized by a concentration of 10^7^ CFU/mL, was introduced in conjunction with 100 μL of *E. coli* phage at varying concentrations ranging from 10^5^ PFU/mL to 10^9^ PFU/mL. The phage was administered at MOIs of 0.01, 0.1, 1, 10, and 100. The pork samples were incubated in an incubator maintained at a temperature of 25°C. At specified intervals of 0, 1, 3, 6, 9, and 12 h, the pork slices were subjected to thorough agitation in 5 mL of sterile PBS for a period of 5 min, after which 20 μL was extracted for inoculation onto LB solid medium plates. The plates were uniformly agitated prior to sampling, with three replicates conducted for each time point, and subsequently incubated at 37°C for 8 h prior to the enumeration of bacterial colonies.

## 3 Results

### 3.1 Pathogenic *E. coli* strain isolation and identification

A strain of *E. coli* was successfully isolated from both a pig farm and a slaughterhouse, with one particular strain designated as EC2318, which serotype is O157:H7.

### 3.2 Antibiotic resistance and virulence genes

**Figure 1:**
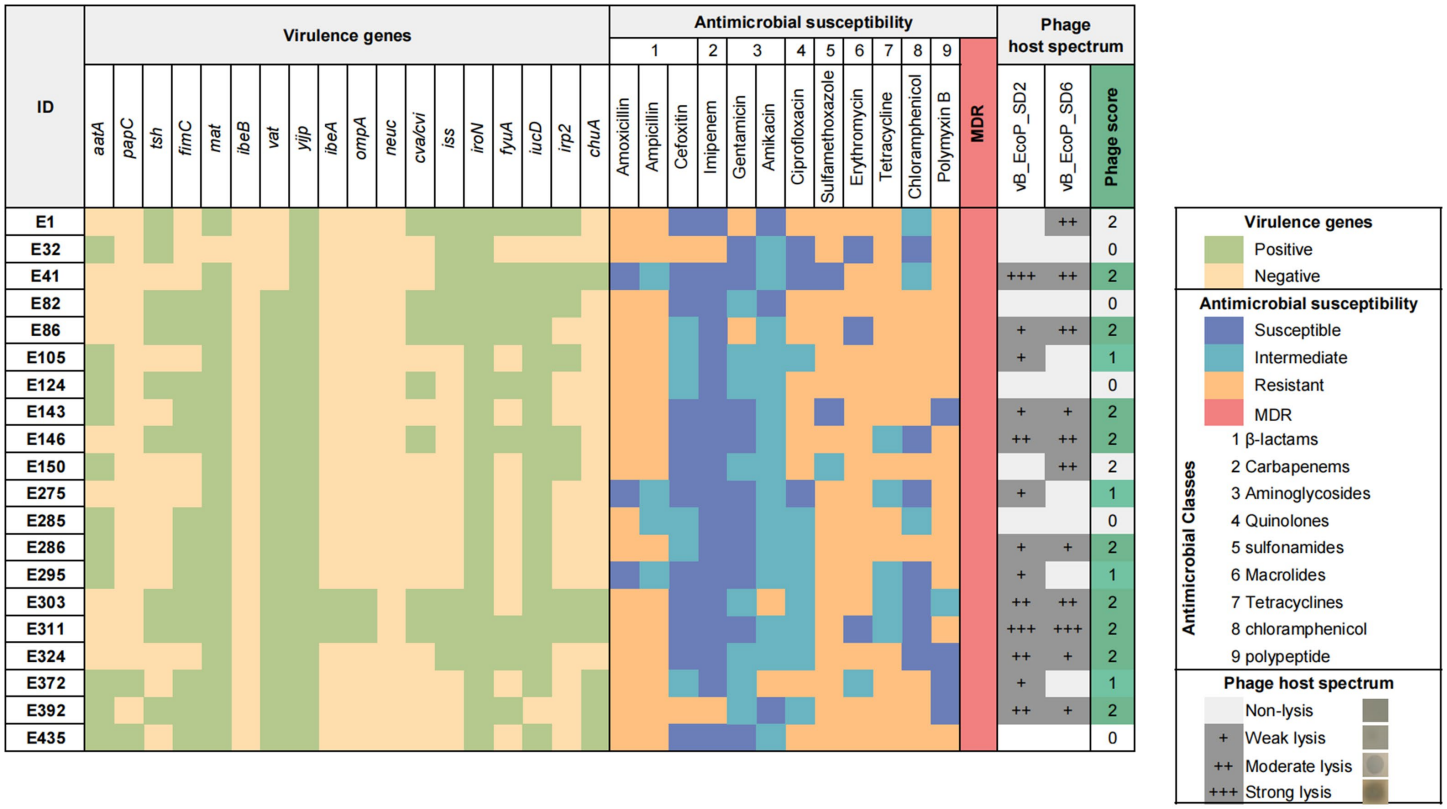
Characteristics and background information of *Escherichia coli* strains and phage host range.

According to the genetic identification results of *E. coli* strains (Figure 1), amoxicillin (85%, 17/20) and sulfamethoxazole (85%, 17/20) had the highest drug resistance rates. Ampicillin (80%, 16/20) and erythromycin (80%, 16/20) had high resistance rates. The drug with the highest sensitivity was imipenem (90%, 18/20). Twenty strains (100%, 20/20) were multidrug resistant. All strains (100%, 20/20) carried *yijp* and *iroN* virulence genes. Most strains carry virulence genes for *mat* (95%, 19/20), *iucD* (90%, 19/20) and *vat* (85%, 17/20).

Heatmap of virulence genes and antibiotic resistance of *E. coli* and lytic spectrum of phages. The heatmap displays the presence of 18 virulence genes in each bacterial isolate, as well as their antimicrobial profiles and 12 antibiotics across 9 classes. The grayscale matrix on the right illustrates the host range of each isolated phage, indicating the plaque clarity from weak lysis (+) to strong lysis (+++). The number of phages to which each bacterial isolate is susceptible is represented as the phage score, reflecting the high combined lytic activity of the two isolated phages.

### 3.3 *E. coli* phages isolation and morphological characteristics

**Figure 2:**
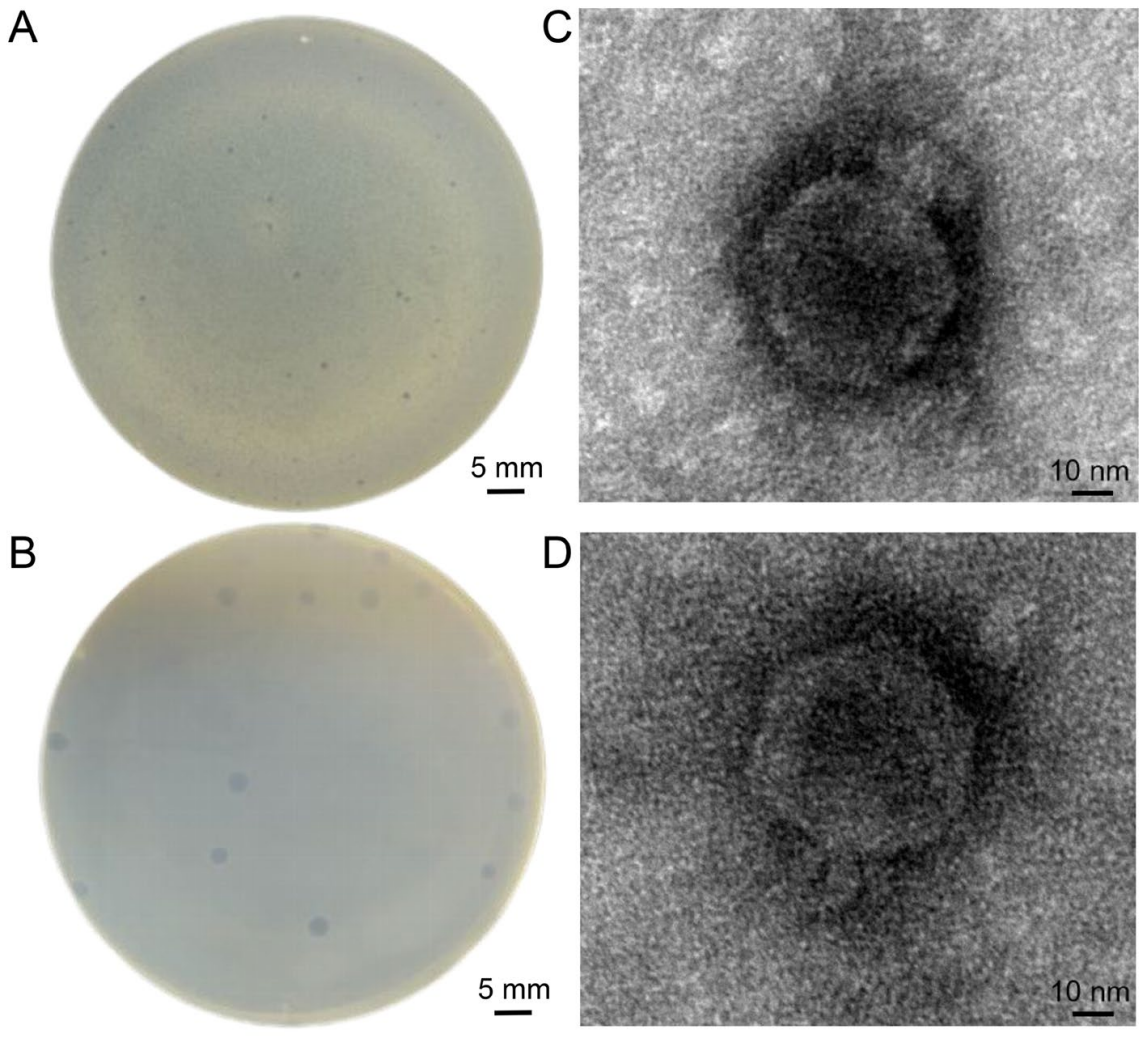
Morphological characterization of phages. **(A,B)** Clear, circular plaques produced by phages vB_EcoP_SD2 and vB_EcoP_SD6 on *E. coli* lawns. **(C,D)** TEM images of phages.

Two phages were isolated from sewage and fecal matter and demonstrated consistent size and plaque morphology when examined on a double-layer agar plate. They were designated vB_EcoP_SD2 and vB_EcoP_SD6. vB_EcoP_SD2 displayed a bright appearance devoid of a halo, measuring 0.3 ± 0.05 mm in diameter by vernier calipers (Figure 2A). In contrast, vB_EcoP_SD6 was transparent and also lacked a halo, with a diameter measuring 0.6 ± 0.05 mm (Figure 2B). Transmission electron microscopy revealed that both phages possess short tails, classifying them within the *Podoviridae* family. Phage vB_EcoP_SD2 exhibited a nearly circular head with a diameter of 60 ± 1 nm (Figure 2C), while phage vB_EcoP_SD6 displayed a hexagonal head with a diameter of 67 ± 1 nm (Figure 2D). The titer of phage vB_EcoP_SD2 was determined to be 9 × 10^8^ PFU/mL, while the titer of phage vB_EcoP_SD6 was found to be 6 × 10^8^ PFU/mL.

### 3.4 Phage lysis spectrum analysis

Twenty pathogenic *E. coli* strains were randomly selected from the laboratory strain collection and subjected to analysis to determine their susceptibility to phages (Figure 1). The results indicated that phage vB_EcoP_SD2 lysed 13 pathogenic *E. coli* strains, representing 65% of the total (13/20), while phage vB_EcoP_SD6 lysed 11 strains, accounting for 55% of the total (11/20). Their complementary host range was higher at 75% (15/20).

### 3.5 Optimal condition for phage infection complex

**Figure 3:**
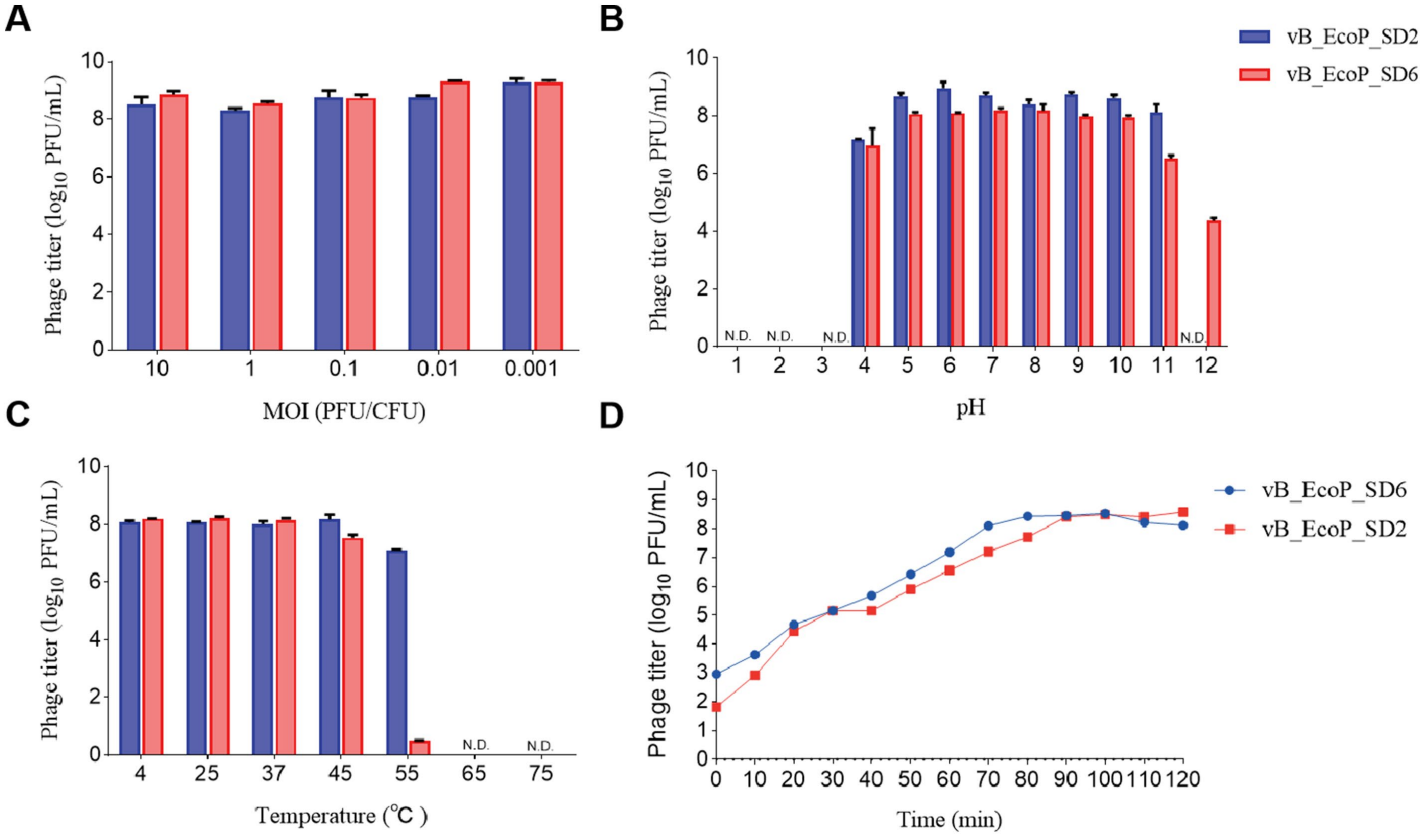
Biological characteristics of phages. **(A)** Influence of different MOI on phage efficacy. **(B)** pH stability of phages across a range of pH levels (1, 2, 3, 4, 5, 6, 7, 8, 9, 10, 11, and 12). **(C)** Thermal stability of phages at various temperatures (4, 25, 37, 45, 55, 65, and 75°C). **(D)** One-step growth curves of phages at optimal MOI over a 2-h period.

The results pertaining to the determination of the optimal MOI are illustrated in Figure 3A. Phage vB_EcoP_SD2 demonstrated the greatest efficacy at an MOI of 0.1. Therefore, the optimal MOI for phage vB_EcoP_SD2 has been determined to be 0.1. In the case of phage vB_EcoP_SD6, the maximum efficacy was observed at an MOI of 0.01, indicating that the optimal MOI for phage vB_EcoP_SD6 was 0.01.

### 3.6 Determination of phage growth kinetics

The outcomes of the one-step growth curve for the phages vB_EcoP_SD2 and vB_EcoP_SD6 are illustrated in Figure 3B. In the case of phage vB_EcoP_SD2, the interval spanning from 0 to 10 min was identified as the latency phase, which was characterized by a relatively stable phage titer. Between 10 and 90 min, a significant increase in phage numbers was observed, reaching an approximate density of 80 PFU per cell. The subsequent period from 90 to 120 min represents a plateau phase, during which the phage titer exhibited minimal fluctuation. Similarly, the incubation phase for phage vB_EcoP_SD6 was designated to span from 0 to 10 min. The phage population exhibited a rapid increase from 10 to 70 min, entering the burst phase with an average density of 10 PFU per cell. The subsequent 50 min were identified as a plateau phase, during which the phage titer exhibited minimal fluctuation.

### 3.7 Phage thermal stability assessment

The results of the thermal stability assessment for phages vB_EcoP_SD2 and vB_EcoP_SD6 are presented in Figure 3C. The phage vB_EcoP_SD2 demonstrated considerable efficacy and exhibited notable stability when subjected to treatment temperatures within the range of 4–45°C. An elevation in temperature to 55°C resulted in a decline of the phage titer to approximately 1 × 10^7^ PFU/mL; upon surpassing 65°C, the phage titer ultimately diminished to zero.

The phage vB_EcoP_SD6 displayed notable efficacy and demonstrated considerable stability when subjected to thermal conditions spanning a range of 4–37°C. Upon exposure to a treatment temperature of 45°C, the efficacy of phage vB_EcoP_SD6 began to decline, reaching an approximate concentration of 3.6 × 10^7^ PFU/mL. Upon elevation of the temperature to 55°C, the phage titer exhibited a precipitous decline, reaching a concentration of 35 PFU/mL. At thermal conditions exceeding 65°C, the titer was rendered at zero.

### 3.8 Phage pH tolerance analysis

The results of the acid–base stability assessment for the phages vB_EcoP_SD2 and vB_EcoP_SD6 are presented in Figure 3D. The phage vB_EcoP_SD2 exhibited notable biological activity across a pH range of 4.0–11.0, with minimal efficacy observed at 1.5 × 10^7^ PFU/mL at pH 4.0 and exceeding 1.5 × 10^9^ PFU/mL when the pH was maintained between 5.0 and 11.0. In environments with extreme acidity (pH 3) and extreme alkalinity (pH 12), the titer of phage vB_EcoP_SD2 was observed to decline to 0 PFU/mL, indicating that it is not viable under such harsh conditions. The data demonstrated that the phage vB_EcoP_SD2 was not resistant to highly acidic or alkaline environments. However, it was stable within the pH range of 4.0–11.0.

The efficacy of phage vB_EcoP_SD6 was demonstrated at a range of pH levels, spanning from 4.0 to 10.0, with a recorded minimum potency of 2 × 10^7^ PFU/mL. At a pH measurement of 11.0, a decline in phage activity was observed, resulting in a reduction in potency to approximately 3.9 × 10^6^ PFU/mL. At a pH of 12.0, the phage titer exhibited a further decline, reaching 2.3 × 10^4^ PFU/mL. In conditions with a high acidity level (pH 3), the phage titer was recorded as 0 PFU/mL. The results indicated that phage vB_EcoP_SD6 demonstrated greater resilience to alkaline environments in comparison to acidic conditions.

### 3.9 Phage genome analysis

**Figure 4:**
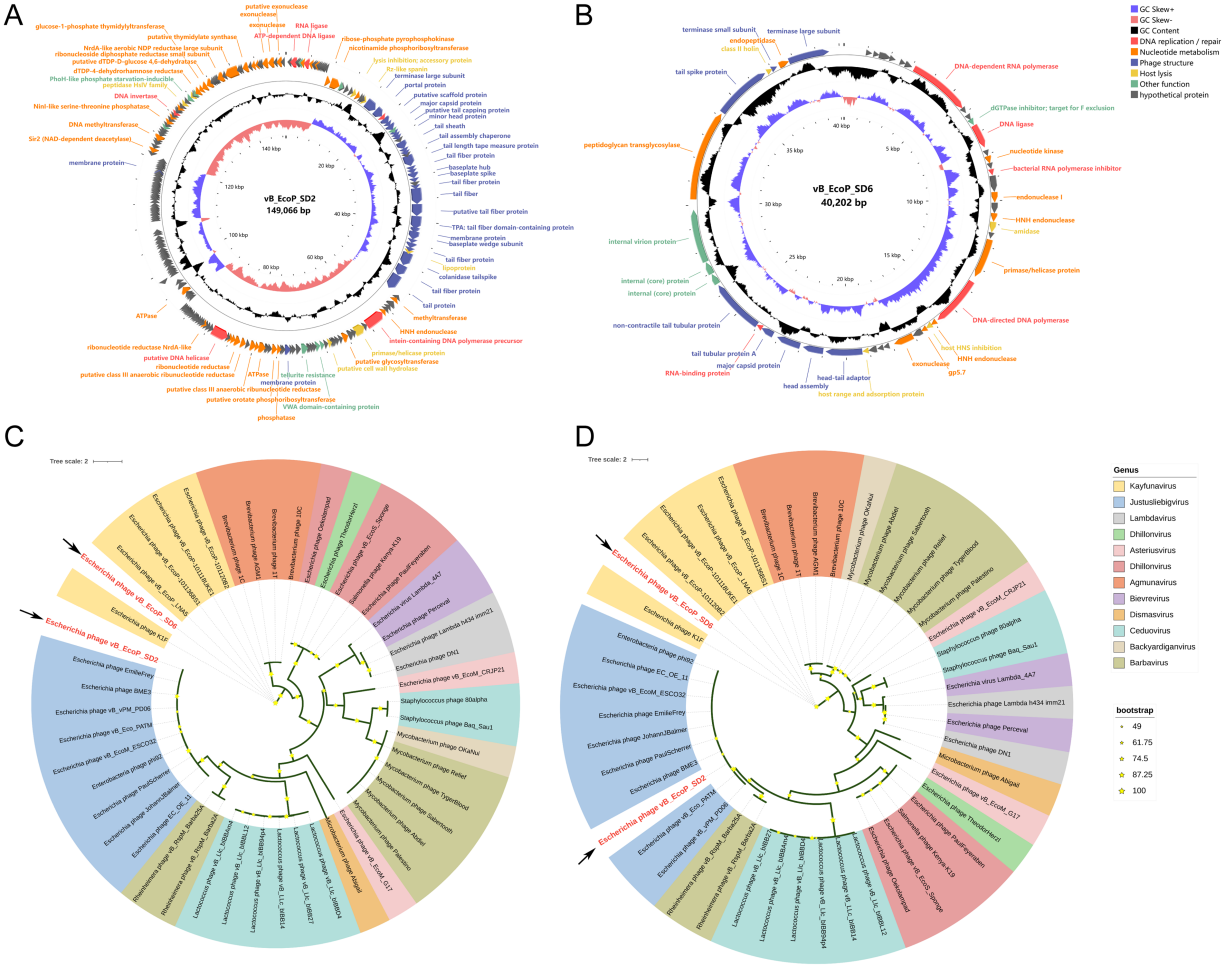
Circular genome maps of phages and phylogenetic trees. **(A)** Circular genome maps of phages vB_EcoP_SD2. **(B)** Circular genome maps of phages vB_EcoP_SD6. **(C)** Phylogenetic trees based on the major capsid protein of phages vB_EcoP_SD2 and vB_EcoP_SD6. **(D)** Phylogenetic trees based on terminase large subunit of phages vB_EcoP_SD2 and vB_EcoP_SD6.

The results of phage genome sequencing are displayed in Figure 4. The original genomes of phage vB_EcoP_SD2 and phage vB_EcoP_SD6 have been uploaded to the NCBI Gene bank database, which login number, respectively, were SRR30892831 and SRR30893832. Through whole-genome sequencing, we found that the genome of phage vB_EcoP_SD2 consisted of 149,066 bp with a GC content of 37.5% and vB_EcoP_SD6 consisted of 40,204 bp with a GC content of 49.8%. The genome analysis identified 244 ORFs, of which 154 encoded hypothetical proteins, and 90 were annotated with known functions. These functions were grouped into five distinct modules, reflecting the phage’s genetic architecture and potential applications (Figures 4A,B).

The replication and regulatory module contained six genes critical for DNA replication and repair processes. In particular, ORF 2 encoded an ATP-dependent DNA ligase, while ORF 7 encoded RNA ligase activity. ORF 31 encoded a DNA methyltransferase, and ORF 70 contained an intein within a DNA polymerase precursor. In addition, ORF 110 and ORF 209 were involved in DNA helicase and invertase activities, respectively. The nucleotide metabolic function module included 37 genes essential for nucleotide metabolism, including key genes such as ATPase and ribonucleoside diphosphate reductase small subunit. This module underscored the phage’s ability to support its metabolic needs during replication. Structural components of the phage were encoded by 31 genes within the phage structural module. These genes were critical for the formation of tail fiber proteins, virion structural proteins, and a putative scaffolding protein, all of which were integral to the physical architecture of the phage. The host lysis module, consisting of seven genes, was dedicated to the lytic cycle of the phage. This module included genes involved in lysis inhibition, accessory proteins, and enzymes such as peptidase HslV family enzymes, which were essential for the phage’s interaction with its host. Lastly, the other functions module contained nine ORFs with diverse roles, including chemotaxis, tellurite resistance, and proteins with thioredoxin domains. These functions contributed to the phage’s adaptability and potential for various applications. The detailed genomic organization highlighted the genetic safety of phage vB_EcoP_SD2 and provided insights into its potential use in biotechnology and therapeutics.

Functional annotation of the protein-coding genes was performed employing BLASTn homology alignment analysis tool in the NCBI database (Supplementary Tables S2, S3). We analyzed the major capsid protein (vB_EcoP_SD2-ORF35, vB_EcoP_SD6-ORF35) and the terminase large subunit (vB_EcoP_SD2-ORF30, vB_EcoP_SD2-ORF47) of two phages, which the phylogenetic relationship between the main capsid protein and terminal enzyme large subunit of two phages and the corresponding two proteins of other 46 phages (Figures 4C,D). The phage vB_EcoP_SD2 is closely related to 9 strains of *Justusliebigvirus* in the same clough, but is far related to 11 other phages. However, vB_EcoP_SD6 is closely related to 5 phages of the *Kayfunavirus* genus, located in the same clade, but is distantly related to 11 others.

### 3.10 *In vitro* analysis of phage-mediated bacterial inhibition

**Figure 5:**
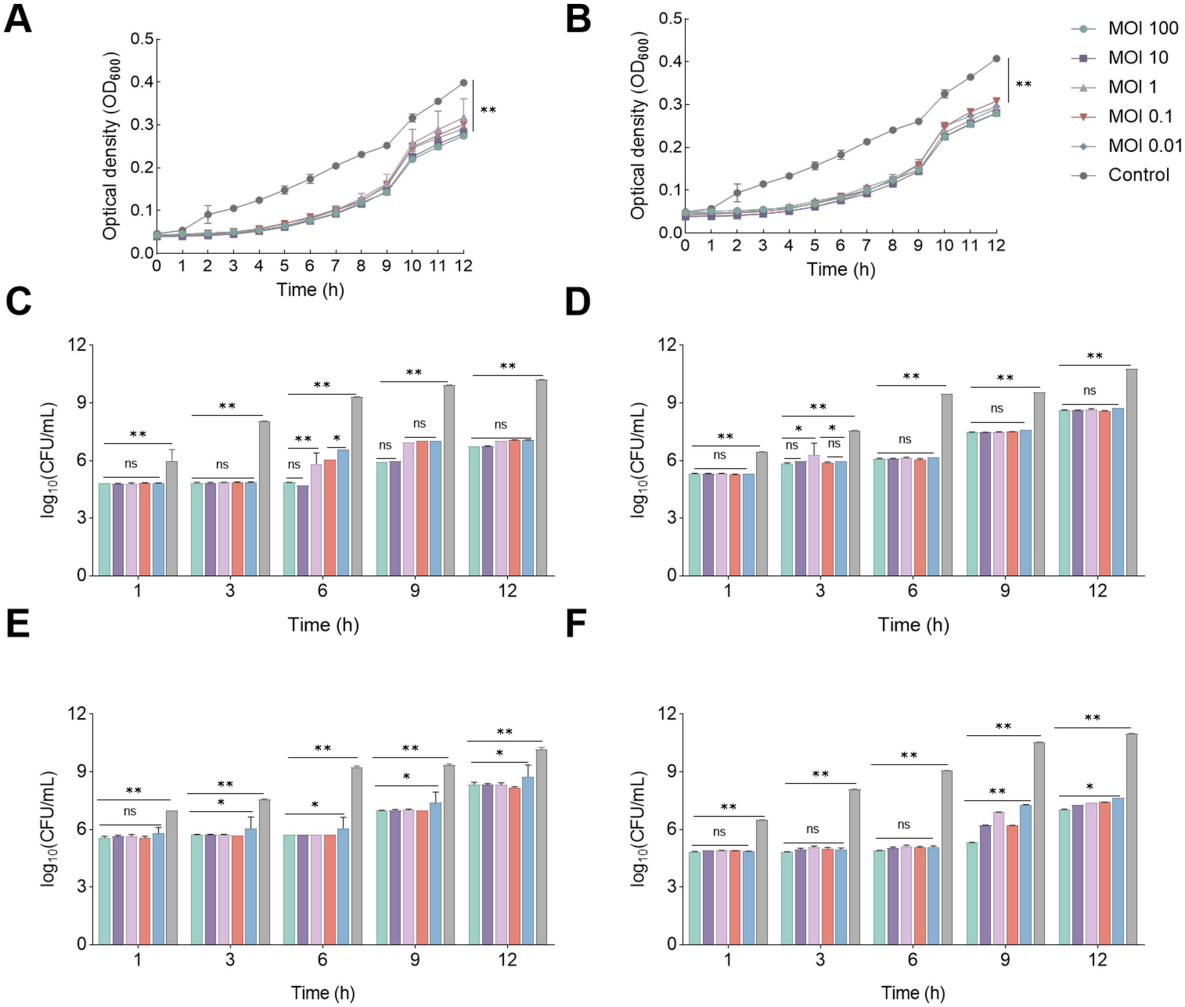
Phage inhibition experiment *in vitro* and phage inhibition experiment on milk and pork. **(A)** Phage vB_EcoP_SD2 inhibition *in vitro* under different MOI conditions. **(B)** Phage vB_EcoP_SD6 inhibition *in vitro* under different MOI conditions. **(C)** Bacteriostasis of phage vB_EcoP_SD2 against *E. coli* in milk under different MOI conditions. **(D)** Bacteriostasis of phage vB_EcoP_SD6 against *E. coli* in milk under different MOI conditions. **(E)** Bacteriostasis of phage vB_EcoP_SD2 against *E. coli* in pork under different MOI conditions. **(F)** Bacteriostasis of phage vB_EcoP_SD6 against *E. coli* in pork under different MOI conditions. *p < 0.05; **p < 0.01, ns *p* > 0.05.

The growth curve of the host bacteria, when co-cultured with phage vB_EcoP_SD2 (Figure 5A) and vB_EcoP_SD6 (Figure 5B) at various MOI. The optical density at 600 nm following the addition of the phage was lower than that of the control group for the initial 5 h, during which the OD_600_ remained essentially constant, indicating a near-complete inhibition of the host bacteria’s growth. After 6 h, the OD_600_ began to increase but remained 0.1–0.12 units below the control group until 12 h. This observation suggested that while phage vB_EcoP_SD2 and vB_EcoP_SD6 potently inhibited the host bacteria, it did not completely suppress their growth.

### 3.11 Phage application in milk bacterial inhibition

The introduction of phage vB_EcoP_SD2 into milk at MOI of 100, 10, and 1 resulted in a negligible change in the number of *E. coli* over the initial 6 h (Figure 5C). However, at the 6-h mark, the count of *E. coli* exhibited a significant decrease of 4.46, 4.59, and 3.7 lg CFU/mL, respectively, in comparison to the control group of host bacteria. These findings suggested that the phage demonstrated robust bacteriostatic efficacy against the host bacteria across all tested MOI during the initial 6-h period. From 6 to 9 h, the number of *E. coli* in the experimental groups with MOIs of 0.1 and 0.01 increased, while the groups with MOIs of 100 and 10 showed minimal change. At the 9-h mark, the count in these groups exhibited a significant decrease of 4.02 and 3.96 lg CFU/mL, respectively, in comparison to the control group. Between 9 and 12 h, the quantity of *E. coli* in all experimental groups increased, yet it remained substantially lower than that in the host bacteria control group.

In terms of phage vB_EcoP_SD6, there was no significant difference between different MOI of phage in each group during the first 6 h of action, but there was a significant difference between them and the bacterial control group (Figure 5D). In the first 9 and 12 h of the experiment, the bacterial proliferation of the phage group was different from that of the first 6 h, with a difference of 1.4 lg CFU/mL, but it was still different from that of the bacterial control group.

### 3.12 Phage-mediated bacteriostatic ability in pork meat

The bacteriostatic capability of phage vB_EcoP_SD2 on pork meat surfaces is depicted (Figure 5E). At 25°C, an increase in the number of *E. coli* in each group was observed over time. Following the addition of the phage to pork meat samples, the number of *E. coli* remained almost unchanged at MOIs of 100, 10, 1, 0.1, and 0.01 for the first 6 h. However, at the 6-h mark, the count exhibited a significant decrease of 4.15, 4.06, 3.95, 4.05, and 4.1 lg CFU/mL, respectively, in comparison to the host bacteria control group. Following the 6-h period, the number of *E. coli* in the experimental groups increased but remained substantially lower than that in the host bacteria control group until the 12-h mark. The bacteriostatic ability of phage vB_EcoP_SD6 on pork surface was shown (Figure 5F). At 25°C, an increase in the number of *E. coli* in each group was observed over time. After the addition of phages to the pork samples, the number of *E. coli* remained essentially unchanged for the first 6 h, with MOI of 100, 10, 1, 0.1, and 0.01, respectively. However, at 6 h, the count decreased compared to the host bacteria control group, with 3.55, 3.59, 3.47, 3.5, and 3.31 lg CFU/mL, respectively. After 6 h, the number of *E. coli* in the experimental group increased, but until 12 h, the number of *E. coli* in the experimental group was still lower than that of the host bacteria control group. This observation suggested that phage vB_EcoP_SD2 and vB_EcoP_SD6 exhibited a potent bacteriostatic effect on *E. coli* populations on pork meat surfaces, with a delayed but substantial reduction in bacterial counts at all tested MOIs.

The antibacterial experiment (Figures 5E,F) further illustrated that following the introduction of phage and host bacteria into pork meat samples at various MOIs, the inhibitory effectiveness at MOI 1, 0.1, and 0.01 was markedly inferior in comparison to MOI 100 and 10. This finding indicated that the efficacy of phage in suppressing bacterial growth was contingent upon the MOI, which was consistent with the findings from the milk study.

## 4 Discussion and conclusion

*Escherichia coli* represents a significant zoonotic pathogen, and the identification of antibiotic-resistant *E. coli* in livestock and poultry operations, alongside meat products, is a common occurrence (Wang et al., 2020). *E. coli* O157: H7 is a common zoonotic pathogen worldwide, which can contaminate food and cause foodborne diseases (Li et al., 2021). As naturally occurring biological inhibitors derived from diverse sources, phages can enhance food safety by eradicating foodborne pathogens when employed as antibacterial agents. Consequently, lytic phages have once again emerged as a promising alternative strategy for the management of pathogenic bacteria (Ghosh et al., 2019; Kakasis and Panitsa, 2019). The potential of phage therapy as a therapeutic modality is currently being investigated, and the continued advancement of this approach requires the biological characterization of phages, encompassing their host specificity and adaptation to bacterial hosts (Amarillas et al., 2017; Sváb et al., 2018; Hyman, 2019).

In the present investigation, we successfully isolated and purified two lytic phages targeting *E. coli* from slaughterhouse sewage, designated as vB_EcoP_SD2 and vB_EcoP_SD6. Both vB_EcoP_SD2 and vB_EcoP_SD6 demonstrated a specific capability to lyse *E. coli*. The lytic efficacy of the two phages was demonstrated by achieving lysis rates of 65 and 55%, respectively, which exhibited enhanced lytic efficacy in comparison to the *E. coli* phages previously isolated by Xu et al. (2018) and González-Villalobos et al. (2021). Our findings indicated that the thermal stability of phages vB_EcoP_SD2 and vB_EcoP_SD6 was optimal within the temperature range of 4–55°C. However, when exposed to temperatures exceeding 65°C, the phage titer diminished to zero, indicating that neither phage exhibited resistance to elevated thermal conditions. This result aligned with the observations recorded for phage *KFS-EC* at 50°C (Lee et al., 2020). Moreover, we observed that vB_EcoP_SD2 exhibited stability across a pH range of 4–11, while vB_EcoP_SD6 demonstrated stability from pH 4 to 10, thus highlighting their resilience to extreme environmental conditions. It is noteworthy that vB_EcoP_SD2 exhibited a greater capacity for resilience to fluctuations in pH compared to vB_EcoP_SD6. Consequently, we ascertained that the stability spectrum of these two phages encompassed the temperature and pH parameters that were commonly encountered in the production, processing, transportation, and storage of food products. As a result, we inferred that these phages possessed the potential to exert bactericidal effects throughout various stages, thereby contributing to the enhancement of food shelf-life and the mitigation of food spoilage and bacterial contamination risks.

The optimal MOI for phage vB_EcoP_SD2 was determined to be 0.1, while the optimal MOI for vB_EcoP_SD6 was 0.01. These findings indicated that both phages were capable of exerting potent antibacterial effects at relatively low concentrations. The latency period for vB_EcoP_SD2 was observed to be 10 min, followed by a burst period at 80 min, with a burst size of 80 PFU per cell. This was distinct from the findings reported by Zhou et al. (2015), who observed a latency period of 30 min and a burst size of 79 PFU per cell for phage JS09. vB_EcoP_SD6 exhibited a latent period of 10 min and a burst period at 60 min, resulting in a burst size of 10 PFU per cell. The latent periods and burst sizes of these two phages were moderate, aligning with the desirable characteristics for biological agents in clinical applications, particularly for vB_EcoP_SD2. Our observations indicated that a short latent period coupled with a large burst size can rapidly kill bacteria. However, we also noted the potential risk that rapid lysis during treatment could lead to the release of endotoxins and possible septic shock.

For the safe and reliable application of phages, genome analysis is essential (Zhou et al., 2015). In the complete genome of phages vB_EcoP_SD2 and vB_EcoP_SD6, no toxin genes, pathogenicity genes, or resistance genes were discerned through examinations of online repositories, suggesting their safety in clinical utilization as bacterial control agents. Genome sequencing facilitated the expeditious identification of deleterious characteristics such as bacterial pathogenicity, resistance, and phage lysogeny-associated genes, permitting the prompt exclusion of unsuitable phages. No genes pertaining to pathogenicity and antibiotic resistance were detected in either phage, indicating that the phages are innocuous to humans at the genomic level. The Justusliebigvirus phage isolated by Markusková et al. (2024) has potential to inhibit bacterial growth during infection, which belongs to the same genus as vB_EcoP_SD2 isolated in this study and they all have significant anti-infection effect. The cleavage rate of *E. coli* phage Kayfunavirus ZH4 isolated by Li et al. (2022) was 11.8% (2/17) and the temperature tolerance was 4–50°C. However, vB_EcoP_SD6 isolated in this study also belongs to the Kayfunavirus genus, but its cleavage rate and temperature tolerance are better than that of *E. coli* phage Kayfunavirus ZH4.A novel lytic phage vB_CtuP_B1 isolated by Zeng et al. (2021) belongs to the genus Kayfunavirus, which inhibited the growth of *C. turicensis*. This is highly similar to vB_EcoP_SD6 in terms of species and bacteriostatic effect.

In 0–12 h of experiment, the number of *E. coli* in the phage group was lower than that in the bacterial control group, indicating that phage has a significant antibacterial effect. The two phages had good effects on contaminated milk and contaminated pork in the first 6 h, but after 6 h *E. coli* began to proliferate, indicating that the bacteriostasis effect of phage decreased after 6 h. The application of phage vB_EcoP_SD2 and vB_EcoP_SD6 to milk resulted in a notable bacteriostatic effect across all five groups with varying MOIs within the initial 6 h. This effect may have been attributed to the phage’s inhibitory action on bacterial growth at the early stage. After 6 h, the bacterial populations in the three experimental groups exhibited a notable increase, with the exception of the groups with MOIs of 100 and 10, potentially attributable to the lower concentration of phages relative to the other groups. However, the MOI groups at 10 and 100 exhibited notable bacteriostatic effects within the initial 9 h, potentially due to the high concentration of phages leading to an excessive cleavage of bacteria in the early stage, resulting in a diminished bacterial concentration. In the bacteriostatic experiment with pork, similar patterns observed in the milk experiment were also evident. A comparison of the bacterial counts in milk and pork revealed that the phage exhibited a superior antibacterial effect in milk. This may have been attributed to the fact that the phage was more prone to binding with the corresponding host bacteria in a liquid medium, thereby exerting its effect on the host bacteria in a more expeditious manner. Abuladze et al. (2008) investigated the efficacy of a phage mixture in reducing the levels of *E. coli* O157:H7 in food samples. Their findings corroborated those of our study, demonstrating that the phage mixture can diminish the bacterial load. In a separate study, Bigot et al. (2011) applied the newly isolated phage *FWLLm1* to artificially contaminated ready-to-eat chicken breast rolls and observed a significant bactericidal effect, with a 2.5 lg CFU/cm^2^ reduction in pathogen concentration. These findings were comparable to those of the present study. Furthermore, Wang et al. (2017) applied the phage for the prevention and control of *E. coli* O157:H7 in fruits and vegetables, achieving positive outcomes. The safety of phages as biological control agents for bacteria is a widely recognized principle among scholars worldwide. The U.S. Food and Drug Administration (FDA) has designated phage products as Generally Recognized as Safe (GRAS), thereby affirming their safety for use.

In the present investigation, two novel phages specific to *E. coli*, designated vB_EcoP_SD2 and vB_EcoP_SD6, were successfully isolated. These phages exhibited commendable tolerance to varying pH levels, robust thermal resistance, and high burst sizes, which remained active over a pH range of 4.0–11.0, and at incubation temperatures up to 55°C for 60 min, indicating wide applicability for food processing and storage. Moreover, the phages vB_EcoP_SD2 and vB_EcoP_SD6 exhibited robust lytic capabilities, and the host range of these two phages are complementary to each other. Comprehensive whole-genome analysis confirmed their safety for prospective therapeutic applications. Genomic comparisons suggest vB_EcoP_SD2 and vB_EcoP_SD6, respectively, constitutes a novel member of a new genus, *Justusliebigvirus* genus and *Kayfunavirus* genus. The *in vitro* assays conducted on milk and pork primarily focused on the two phages. At MOI ratios of 10 and 100, notable bactericidal efficacies against *E. coli* were observed in both milk and pork within a span of 9 h. These phages have the potential to function as novel *E. coli* biological inhibitor, thereby providing a theoretical framework for the advancement of phage-based bacteriostatic agents within the food industry.

## Data Availability

The original contributions presented in the study are publicly available. This data can be found at: https://www.ncbi.nlm.nih.gov/sra/, BioProject PRJNA1167002, accession numbers SRR30892831 and SRR30893832. The GenBank accession numbers of vB_EcoP_SD2 and vB_EcoP_SD6 are PQ821640 and PQ821641.
