# Supplementary material for: Biological characterization of novel Escherichia coli O157:H7 phages and their bacteriostatic effects in milk and pork
Source: Front Microbiol. 2025 Feb 6;16:1516223. doi: 10.3389/fmicb.2025.1516223 (PMC11841896; doi:10.3389/fmicb.2025.1516223)
Supplement: Supplementary file 3 [file Data_Sheet_1.PDF]

Supplementary Table1 Primers sequence information of virulence genes

Virulence gene	Primer name	Primer sequence	Destination strip length /bp
Adhesion related genes	aatA-F	CATAGGCGTTTCTCTTTCCGAT	1226
	aatA-R	CCTGTCGTTCATACAGATTCGTT	
	papC-F	GCTGATATCACGCAGTCAGT	768
	papC-R	GTCAACAAGAAGACGTGTTCC	
	tsh-F	GTCTGTCAGACGTCTGTGTTTC	598
	tsh-R	ATAGGATGACAGGCTACCGAC	
	fimC-F	GCCGATGGTGTAAAGGATGG	475
	fimC-R	GGGTAAGTGCGCCATAATCA	
	mat-F	CGACCTGGTCAGCAACAGCC	238
	mat-R	TCCACGCCCACATTCAGTGT	
Invasion and toxin related genes	ibeB-F	GTTCTCACTCAGCCAGAACG	1172
	ibeB-R	CATCCAGCACTTCCAGATAAC	
	vat-F	TCCATGCTTCAACGTCTCAGAG	939
	vat-R	CTGTTGTCAGTGTCGTGAACG	
	yijp-F	TGGCTTGATTCTGCATCCGAT	517
	yijp-R	CATCGTCTGCTGGTTGGTGAT	
	ibeA-F	GTATGACGGTGGGAACAAGAG	321
	ibeA-R	TGGCAATAGCAGCGGCAGTC	
Antiserum survival factor related gene	ompA-F	AGCTATCGCGATTGCAGTG	919
	ompA-R	GGTGTTGCCAGTAACCGG	
	neuC-F	GGTGGTACATTCCGGGATGTC	792
	neuC-R	CATGGTGGTGAAAAGACATTAGC	
	cva/cvi-F	TCCAAGCGGACCCCTTATAG	598
	cva/cvi-R	CGCAGCATAGTTCCATGCT	
	iss-F	ATCACATAGGATTCTGCCG	309
	iss-R	CAGCGGAGTATAGATGCCA	
Iron transport-related genes	iroN-F	CCTCCGACGATGATAATGACG	866
	iroN-R	GATACCATTATGCGTAATGCC	
	fyuA-F	ATGTGAAACTGCGTCTGGCG	728
	fyuA-R	CGCAGTAGGCACGATGTTGTA	
	iucD-F	GAAGCATATGACACAATCCTG	613
	iucD-R	CAGAGTGAAGTCATCACGCAC	
	irp2-F	CTGATGAACTCACTCGCTATCC	440
	irp2-R	AGCATCTCCTGGCTCTGCTC	
	chuA-F	GACGAACCAACGGTCAGGAT	278
	chuA-R	TGCCGCCAGTACCAAAGACA	

Amplification system and amplification procedure:

Adhesion-related gene multiplex PCR amplification system: 2×Taq PCR Mix 12.5 μL, DNA

template 1 μL, aatA, tsh upstream and downstream primers 0.75 μL each, papC, fimC, mat upstream and downstream primers 0.5 μL each, add ddH2O to make up to 25 μL ; Invasion and toxin-related gene multiplex PCR amplification system: 2×Taq PCR Mix 12.5 μL, DNA template 1 μL, ibeB upstream and downstream primers 0.75 μL each, vat, yijp, ibeA upstream and downstream primers 0.5 μL each, add ddH2O to make up to 25 μL; antiserum survival factor-related gene multiplex PCR amplification system: 2×Taq PCR Mix 12.5 μL, DNA template 1 μL, ompA, neuC and cva/cvi upstream and downstream primers 0.5 μL each, iss upstream and downstream primers 1 μL each; iron Transport-related gene multiplex PCR amplification system: 2×Taq PCR Mix 12.5 μL, DNA template 1 μL, iroN, fyuA, iucD, irp, chuA upstream and downstream primers 0.5 μL each, add ddH2O to make up to 25 μL. The concentration of each virulence gene primer is 10 μmoL/L. Each multiplex PCR amplification program was: pre-denaturation at 95°C for 5 min; 30 cycles of denaturation at 94°C for 60 s, annealing at 55°C for 60 s, and extension at 72°C for 60 s; and extension at 72°C for 10 min. After the reaction, take 5 μL of the amplified product for 1.0% agarose gel electrophoresis, observe the results on the gel imaging system and take pictures.
